# Late Termination of Pregnancy. Professional Dilemmas

**DOI:** 10.1100/tsw.2003.81

**Published:** 2003-09-23

**Authors:** Isack Kandel, Joav Merrick

**Affiliations:** Faculty of Social Science, Department of Behavioral Sciences, Academic College of Judea and Samaria, Ariel, DN Ephraim 44837, Israel

**Keywords:** abortion, late abortion, late termination, induced abortion, ethics, religion, disability, handicap, Israel

## Abstract

Abortion is an issue as long as history and hotly debated in all societies and communities. In some societies and countries it is legal, while other countries have no legal basis, and some countries have made it a crime. Today up to 90% of abortions take place in the first trimester, about 9% in the second trimester, and the rest in the third trimester.This paper deals with the issue of late termination of pregnancy, the practical medical aspects, legal issues, international aspects, and the dilemma for the professional.In early history, abortion was accepted by clergy and societies, but in recent history it is more restricted and in some countries prohibited. It does not seem that restriction leads to a lower abortion rate, but rather an active contraceptive policy, campaign, and availability to prevent pregnancies that are unwanted. In countries where abortion is restricted, the trend has been an increase in illegal abortion that leads to unsafe abortion with complications, permanent injuries, and maternal mortality.Unsafe and illegal abortion is a public health concern that governments should try to prevent and instead find ways to strengthen their commitments toward better and safer health and family planning services for women.Late termination of pregnancies is an issue of grave concern with many practical medical aspects, ethical questions, and professional dilemmas. This is especially of concern because of the viability of the fetus and should only take place in order to prevent harm to the physical and mental health of the mother or due to an anomaly or disability of the fetus.

## INTRODUCTION

Abortion is defined as the termination of pregnancy before a fetus is viable. Viability is today considered to be reached at 23–24 weeks of pregnancy, however, some authors claim, on ethical grounds, that the fetus is only viable from 24 weeks and older[1]. Spontaneous and habitual abortion may occur early, before week 16, or late abortion from 16–28 weeks. About 12% of all diagnosed pregnancies terminate in a spontaneous abortion. More than 60% of these result from ovular or fetal defects, while about 15% are caused by maternal factors.

Induced abortion (elective or therapeutic) is the artificial termination of a viable pregnancy, an issue as long as history and hotly debated in all societies and communities. In some societies and countries it is legal, other countries have no legal basis, and some countries have made it a crime.

This paper will deal with the issue of late termination of pregnancy, the practical medical aspects, legal issues, international aspects, and the dilemma for the professional.

## LATE TERMINATION OF PREGNANCY

Late termination, or feticide because the fetus is now viable and could survive outside the womb, means termination of a pregnancy after 24 weeks. This subject is an even more hotly debated issue than early abortion, because the unborn child is viable and we are talking about the killing of an unborn child. Some even go as far as to call it organized murder.

It is estimated that there are at least 26 million legally terminated pregnancies each year throughout the world and at least 20 million illegally terminated, with the result of at least 78,000 maternal deaths[2].

Only about 8-10% of all abortions performed in the United States are performed in the second trimester, but single adolescent females are disproportionally represented[3]. One study from Missouri, United States, examined 97 adolescents aged 13-17 years who came for late termination, looked at risk factors for late help seeking and found that contraceptive use and a positive attitude towards abortion were predictive of late arrival at the abortion clinic[3].

A study from 21 hospitals in a northern province of Holland[4] covering the years 1990–94 showed that 14 hospitals had performed 113 terminations of pregnancies beyond week 24. In 21% of the cases, anencephaly was diagnosed before termination, 21% were diagnosed with severe chromosomal defects, 16% hydrocephalus or spina bifida, 12% no renal function, and 11% with skeletal abnormalities. In 28% of the cases, the pregnancy was terminated at 28 weeks, between week 29–37 in 64% of the cases, and after week 37 in 8%[5].

Late termination has also been studied at the Maternite Port Royal University Hospital in Paris, France[6], where a consecutive series of 956 terminations were studied for the period of 1986–94; 305 were in the third trimester and 651 in the second. In 55 cases (18%) of third-trimester termination, an anomaly could not have been diagnosed before the third trimester, in another 122 (40%) the prognosis of an anomaly could not have been established firmly until third trimester, in 15 cases (5%) the poor prognosis of the fetus was established in the second trimester, but termination postponed for various reasons, and in the final 113 cases (37%) the condition could have been detected earlier in pregnancy, but screenings during the second trimester resulted in false negative results (like myelomeningocele, trisomy 13 and 18, and lethal dwarfism). Another French study was done at the University Hospital of Tours[7], during 1992–95 on 125 females who underwent second- and third-trimester termination. Fetal indications were dominant at 43%, chromosomal anomalies 30%, and maternal indications 3%.

In a study from the University of Bari in Italy[8] during 1988–97 with 330 females, 123 (37%) had termination due to psychiatric disorder due to fetal pathologies and 205 (62%) due to psychiatric disorder linked to the pregnancy itself; 23.3% were teenagers, 50% singles, and 16.4% students.

## PRACTICAL MEDICAL ASPECTS

Today up to 90% of abortions take place in the first trimester and about 9% in the second trimester. If an abortion has to take place, the earlier the better because less complications are involved.

There are different options today to abort an unwanted pregnancy. There is the so-called "morning after pill" or **emergency contraception** (EC)[9], an idea developed by the Canadian professor A. Albert Yuzpe in the 1970s, where two doses of oral contraceptive pills that combine estrogen and progestogen hormones are used 24–72 h after sexual intercourse, when unwanted pregnancy may have occurred. New preparations, like Plan B or levonorgestrel, which only contain progestogen, have fewer side effects. It seems that the method of postcoital contraception has three possible effects preventing implantation — inhibition of ovulation, alternation of the normal menstrual circle, and alternation of the endometrium — or, in other words, a form of chemical abortion.

**RU 486** (Mifeprex or mifepristone) was developed by Etienne Emile Baulieu from France[10] as the first of a new generation of antiprogestin drugs, which bind the progesterone receptors in the uterus and block the action of progesterone, the hormone produced by the female to stimulate uterine glands to prepare the uterus for pregnancy. RU 486 or methotrexate (a drug used in oncology and certain chronic diseases) is used sequentially with misoprostol (a drug used to treat peptic ulcers) as a medical termination of pregnancy (or nonsurgical abortion) up to 49 days after the start of the last menstrual period. Medical termination of pregnancy in the first trimester has been excellently reviewed recently[2] and the reviews show that medical abortion is associated with higher rates of prolonged bleeding, nausea, vomiting, and pain than surgical abortion. The success rate for medical abortion is also lower and it is time consuming for the female and the medical center (three visits usually: one visit to diagnose the pregnancy and receive RU 486, a second visit 48–72 hours later to receive misoprostrol, and a third 2 weeks later to see if the abortion was complete), but acceptable to a majority of women seeking abortion[2].

One study from Aberdeen[11] looked at 483 cases of medical abortion in the late first trimester with the above method and found complete abortion in 458 cases (94.8%) with decreased efficacy with advanced gestational age. A study from Denmark[12], where medical abortion was introduced in 1997, found that medical abortion was performed in 25 out of the 50 departments performing abortions. There were great variations in dose and administration, upper gestational limit, and follow-up procedures, which calls for a need of national guidelines.

**Suction curettage** (or vacuum aspiration) is used in early pregnancy (6–7 weeks). The cervix is dilated or widened and a suction tube inserted into the uterus and connected to vacuum so that the embryo can be removed by suction.

**Dilatation and curettage** (D&C) is used a little later (and also used after miscarriage) and here again the cervix is dilated and an instrument (with a sharp loop in the end) inserted in the uterus, where the inside wall is scraped with the lining and the embryo removed by vacuum or suction.

**Dilation and evacuation** (D&E) is used in the second trimester (8–20 weeks) by inserting laminaria (a seaweed) into the cervix 6–8 h before D&E or prostaglandin $E_2$ ($PGE_2$) in a gel applied to the cervix 12–24 h before in order to soften the cervix and allow easier dilatation. Afterwards, the cervix is dilated to about 5 cm to allow removal of the fetus.

Last trimester or late termination is not common (about 1% of all abortions) because at this stage the fetus is viable and the termination more complicated. There are several methods in use, with the administration of intravenous prostaglandin most widely used (85%)[5]. Here **prostaglandin $F_{2\alpha}$ ($PGF_{2\alpha}$)** is given as an intravenous infusion and induces abortion in 12–19 h, but here there is a chance of the fetus being born alive. In a Dutch study, 80% were born stillborn and the live born infants died within the first 24 h of birth[5].

**Intra-amniotic injection of solutions** will also produce evacuation of the uterus within 12–14 h. Transabdominal aspiration of amniotic fluid is performed with immediate, very slow replacement with a similar amount of sterile aqueous 20% sodium chloride or 50% urea in 5% dextrose and water. Labor will follow (or induced by oxytocin) and a dead fetus delivered.

**Hysterotomy**, either abdominal (like a cesarean section) or vaginal, has the disadvantage of delivering a live fetus, when the pregnancy is advanced and the rate of complications greater.

Another method is **injection of potassium chloride (KCl)** into the fetal heart under ultrasound guidance with one study of ten terminations in week 22–38[13] that showed it to be a safe method for the mother and a painless death of the fetus. In another study by the same group in Poissy, France[14], **lidocaine** (1%) was used in 50 women by umbilical vein puncture under ultrasound guidance with injection of sufentanil (5 µg) followed by 7–30 ml lidocaine. This procedure was successful in 92% of the cases (46/50) with complete cessation of fetal heart activity and no maternal side effects.

In cases where feticide cannot be achieved before delivery, within the framework of late termination of pregnancy, another French group has suggested accompanying the baby to death in the neonatal unit[15].

## SOME LEGAL ISSUES AND INTERNATIONAL ASPECTS

There has been a long debate on the issue of the right of the fetus in maternal-fetal medicine[16] with the argument that the viable fetus[1] should be considered a fetal patient for the physician and the previable fetus only a patient if the pregnant women confers such a status on it.

A review of abortion in law, history, and religion from an international perspective can be found in a pamphlet from "Childbirth by Choice" from 1995[17]. Abortion has taken place throughout history with even Socrates, Plato, Aristotle, and Hippocrates suggesting abortion[17]. The Romans did not place any social stigma on abortion, and early Christians condemned it, but only after the stage of "ensoulment" or "quickening", when the women feels the movements of the fetus[17].

**Canon Law** (the juridical law of the Roman Catholic Church given by the Pope alone or together with a council) and theologians believed that the viability of the fetus began at 40 days for a boy and 80 days for a girl and in the year 1140 accepted that early abortion was not homicide. If abortion was done after "quickening", it was homicide and the person would incur an automatic (latae sententiae) excommunication[17]. This was indeed a tolerant view by the Church that ended in 1869, when pope Pius IX eliminated the distinction and required excommunication for abortions at any stage of the pregnancy[17]. This is the stand of the Roman Catholic Church today.

From 1307 to 1803, abortion before the fetus moved was acceptable and not punished under **English common law** and was accepted by society. In the "Twinlayer's Case" from 1327 and the "Abortionist's Case" in 1348, the judges would not make the death of a fetus a legal offense[17]. In 1670, the English judge Sir Matthew Hale, ruled that an abortionist would be guilty of murder only if the woman died and in 1803, Lord Ellenborough was responsible for the criminal abortion law that stated that the abortion of a "quick fetus" would be a capital offense, while abortion prior would be a lesser offense[17]. This law was modified in 1938 with the "Bourne Case", where the physician Alec Bourne performed an abortion on a 14-year-old girl who had been raped by soldiers. He turned himself in, but was acquitted, because he defended the abortion "because otherwise the pregnancy would have made the women a physical and mental wreck"[17]. In 1967, an act was passed that allowed abortion in England if two physicians found the continuation of the pregnancy a risk to the life of the woman, to her physical and mental health, to any of her existing children, or if the fetus suffered an anomaly. In 1990, the House of Lords passed the Human Fertilization and Embryology Bill to cut the legal time limit for abortions from 28 to 24 weeks of pregnancy, but also removed the upper limit in case of fetal disability[18]. In 1991, medical abortion with RU 486 was approved in England[17].

In the U.S., legislation was similar to the British Law and first trimester abortions legal, but by 1900 were prohibited throughout the states unless two or more physicians agreed that the procedure was necessary to preserve the life of the woman[17]. **American Law** is complex due to the system of federalism, the theory of the separation of powers, and the doctrine of judicial review. Abortion law stems from two basic governmental sources: the legislatures of the several states and territories, and the Supreme Court. In the 1960s, the complications and mortality from illegal abortions forced state legislatures to change the law and by 1973, abortion laws had been liberalized in 17 states[17].

In 1973, in a landmark case, the U.S. Supreme Court (*Roe v. Wade*) ruled that the Fourteenth Amendment to the U.S. Constitution provided a fundamental right for women to obtain abortions. The court ruled that during the first trimester, the state cannot bar any woman from having an abortion from a licensed physician. During the second trimester, the state can regulate the abortion procedure only to protect the health of the woman and in the third trimester, the state may regulate to protect fetal life, but not at the expense of the life or health of the mother[17]. The 1980s and 1990s saw several attempts to overturn the 1973 decision with increased violent behavior from antiabortion groups with culmination in 1993–94 after several murders of physicians who had performed abortions[17].

RU 486 became legal in France in 1988 and is now available in Britain, Sweden, Germany, Greece, Belgium, Finland, and China. The company Roussel-Uclaf manufactured the pill in France, however, the company was unwilling to manufacture the pill for the U.S. when the time came to apply to the Food and Drug Administration (FDA). The FDA issued an alert prohibiting the importation of RU-486 into the U.S. during the Bush administration because of safety concerns they had for women, who might use the pill to induce abortion. During the 1992 presidential election, both candidates took a strong stand on the abortion pill issue. While Bush continued to support the ban on the import of RU 486, Clinton claimed one of his first duties as president would be to begin the process of bringing the abortion pill to the U.S. Three days after Clinton took office in 1992, he signed an executive order to promote the testing, licensing, and manufacturing of the drug in the U.S. Due to several factors, RU 486 was only approved in the U.S. by late year 2000.

In **Canadian Law,** the Parliament passed a criminal law in 1869 that prohibited abortion and carried a life imprisonment sentence. In 1892, a law was also passed that prohibited the sale, distribution, and advertisement of contraceptives. The law was amended in 1969 to legalize contraception and certain abortions performed by a qualified physician in an approved hospital. The issue of abortion has been hotly debated in Canada with the physician Henry Morgentaler in the forefront defending the rights of women to have an abortion in spite of legal and violent opposition. RU 486 has still not been approved in Canada[17].

In **western Europe** induced abortion is legal, except for the Irish Republic, Northern Ireland, and Malta[17]. In **central and eastern Europe**, the Supreme Soviet liberalized abortion laws in 1955, which was followed by all the others in the bloc, except Albania. In the 1970s, restrictions were enacted in Bulgaria, Czechoslovakia, Hungary, and Romania due to a low birth rate, but it should be remembered that eastern Europe for years used abortion as a form of birth control, where the women who had abortions were generally older, married, and already with children. This is changing, but still there are 3–4 million abortions in Russia every year[17].

In the **Middle East**, where Islam is the leading religion, the Koran does not mention abortion and theologians have therefore given different points of view. There is no actual approval of abortion, nor a ban, but the interpretation tends to be conservative and on medical grounds. In Israel up to 1977, abortions were performed on medical grounds, but in 1977 this was liberalized to include family and social circumstances, however, this part was taken out in 1979. Today a review of at least three persons (a physician with specialty in obstetrics and gynecology; a physician with another specialty like family medicine, pediatrics, psychiatry, or public health; and a social worker) can permit abortion under four situations: to a women under 17 or over 40 years; pregnancy as a result of out of wedlock relationship, rape, or incest; suspected physical or mental disability of the fetus; and danger to the physical or mental health of the mother[17].

In **Africa,** only two countries (Togo and Tunisia) have legal abortion on request and two others (Burundi and Zambia) have legalized abortion for health reasons, while the rest have restricted abortion. Many still have the outdated colonial French, Belgian, or English law that has not been updated[17].

In **Latin America and the Caribbean,** the Catholic Church is powerful, yet it is estimated the area has the highest rate of induced abortion with 2–8 million per year. Most countries (except Barbados, Belize, and Cuba) have restrictive abortion laws, which results in illegal and unsafe abortions with high maternal mortality[17].

In **Asia and Oceania** there are many different countries and cultures. In Australia, the laws concerning abortion are the responsibility of each of the eight individual states and territories with laws originally based on English Law. Only South Australia has a law from 1969 legalizing abortion if two physicians agree to the abortion as in the best interest for the mental and social health of the woman. In all other states it is a criminal offense, but in practice there have been no prosecutions since the 1970s[17]. It is interesting that the law in Australia does not recognize fetal abnormality as a reason for abortion, but only on the grounds of maternal health[19].

China has had free abortion on request since 1957, and has been under harsh criticism worldwide since the 1970s due to the policy of “one child”, with forceful abortions and killing of female newborns. India has had legal abortion since 1971, but still about 4 million illegal abortions are performed per year. In Indonesia, abortion was not allowed by the Dutch, but after independence — while technically still illegal — legislation from the 1970s permitted discreet medical abortion. In Japan, contraception is still not effective and abortion has been used as fertility control. A law from 1948 allows abortion. Malaysia, New Zealand, and Vietnam allow abortion, while Pakistan and the Philippines do not[17].

## THE PROFESSIONAL DILEMMA

Abortion, especially the late termination of pregnancy, can be an extremely traumatic event for the female involved, but also for the attendant medical and nursing staff. As we have seen above, there are serious clinical and legal issues involved, but also ethical aspects and dilemmas concerning:

- The female and her partner
- The fetus
- Legal issues in the country
- The hospital or medical center
- The physician, nurse, midwife, or other professional or staff involved
- Registration responsibilities
- Review or ethical committee

The **female** in need of late-termination pregnancy and also the partner involved should receive supportive counseling before, during, and after the procedure. A multidisciplinary approach should be taken, since many ethical, sometimes religious, and other issues will be involved. Modern medicine and technology have made it possible to screen for and detect disease and anomaly at a very early stage of pregnancy. The obstetrician is therefore in a position to detect early, but also has the duty to advise, recommend, give support, and help the parents in a very hard emotional decision-making process[20].

The truly difficult dilemma arises when the **fetus** is viable[1,16,21] and the question becomes: when is the fetus a patient also? Obstetricians from Cornell Medical Center[1,16,21] have struggled with this concept for some years, in the context of their residency program. For them, the fetus is not a patient when it is nonviable (or unless the female grants the fetus that status) and they therefore find that abortion is justified when the pregnant woman consents. On the other hand, when the fetus is viable it becomes a patient and abortion, with very few exceptions, is therefore not justified ethically[21]. They also find that it is not justified to require residents to perform abortions when/if that practice is against their personal conviction.

**Legal issues** have been touched on in the section above from an international perspective, but for each professional involved in abortion it is very important to "know the rules" of the country where he/she is working in order to stay on the right side of the law. Laws change and physicians or other professionals have been instrumental in some of these changes (like Henry Morgentaler in Canada, as an example), which is important because as professionals in the field of medicine, involvement in society and social change is part of medicine. As the famous German physician Rudolf Virchow (1821–1902), one of the forerunners in social medicine, said: "medicine is a social science and politics nothing but medicine on a grand scale"[22,23]. For the professional, it is also important to confer with his/her professional society. In many countries, expert groups have produced guidelines and information concerning late termination of pregnancy (for example, see the website: www.rcog.org.uk of the British Royal College of Obstetricians and Gynaecologists).

In many countries, **hospitals, medical centers, and clinics** are involved in abortion and especially late termination. Here the administration must be very aware of the legal issues involved and try to monitor the performance of the Obstetrics and Gynecology Department. In some countries (like the U.S. and Canada), the public reaction has been very violent resulting in shooting incidents, bombing, or burning down clinics, which certainly has many ethical and security dilemmas for the administration.

The **professionals** involved with abortion have sometimes experienced many ethical and professional dilemmas. One study from Cambridge in the U.K.[24] compared the attitudes of obstetricians in 1980 with those from 1993. Questionnaires were sent to 555 nonacademic obstetricians and the trend showed that more obstetricians in 1993 were willing to perform late termination for fetal abnormality and social reasons, but many were not prepared to recommend termination as late in pregnancy as the law allowed.

One Australian study[25] showed that practitioners in clinical genetics and obstetrical ultrasound found termination at 24 weeks not acceptable and the author argues that fetal abnormality for late termination to be inconsistently applied, discriminatory, and eugenic[25].

In France, it seems that professionals are more positively inclined towards the late termination of pregnancy[26,27]. In one questionnaire study[27] of 109 professionals working in a tertiary obstetrical unit, where late termination was performed routinely, a majority felt that late termination was needed to avoid fetal and neonatal agony and pain.

In the U.K., the law is clear[28] about the right that any physician, nurse, midwife, or any other paramedical staff may refuse to participate in abortions.

Any professional with objections to abortion should be able to turn to their professional or spiritual mentors for advice and not do any actions against their conscience.

Each abortion should be **registered** according to the law in each country. In the U.K., when a fetus is born before the 24th week of gestation and does not breathe or show any signs of life, there is no provision for the event to be registered. However, the physician or midwife who attends the delivery will need to issue a certificate or letter for the funeral director, cemetery, or crematorium stating that the baby was born before the legal age of viability and showed no signs of life to allow a funeral to proceed, if that is the wish of the parents. When a child is born after 24 weeks, but does not breathe or show any sign of life (therefore classified as a stillbirth), these must be registered within 3 months and the Registrar will allow a funeral to proceed. In the event a child is born and shows signs of life but subsequently dies, both the birth and death need to be registered, irrespective of the gestation period of the child, and the Registrar will then issue a form to allow the funeral to proceed. The Registrar General's Office has confirmed that midwives are permitted to certify stillbirths only. In cases where there are any signs of life, the baby must be seen by a medical practitioner and that practitioner must sign the death certificate (otherwise it becomes a Coroner's case). In situations where parents feel they are unable to act as informants to effect a registration, alternative informants, such as those "present at the stillbirth" or "present at the death", are acceptable in order that registration be achieved with as little distress to the family as possible (see the website: www.rcog.org.uk of the British Royal College of Obstetricians and Gynaecologists).

Some countries have established **review or ethical committees** to handle requests for abortion. This is the case in Israel, where abortions are performed in hospitals or medical centers approved by the Ministry of Health[29]. This is also the case in parts of Australia[30] with some variation in the members of the committee. At the Royal Women's Hospital, the members are from the executive: a neonatologist, two medical divisional directors, one nursing divisional director, and the obstetrician managing the case. At Monash Medical Center, the members are the clinicians involved in the case and a family physician from outside the hospital[30]. This trend in some countries can be seen as a need for making important decisions on termination of pregnancy on a multidisciplinary basis and to share the responsibility of decision making in this very sensitive ethical dilemma.

## CONCLUSION

Abortion is as old as the human being. In early history, abortion was accepted by clergy and societies, but in recent history more restricted and in some countries prohibited. It does not seem that restriction leads to a lower abortion rate, but rather an active contraceptive policy, campaign, and availability to prevent pregnancies that are unwanted. In countries where abortion is restricted, the trend has been an increase in illegal abortion that leads to unsafe abortion with complications, permanent injuries, and maternal mortality.

Unsafe and illegal abortion is a public health concern that governments should try to prevent and instead find ways to strengthen their commitments towards better and safer health and family planning services for women.

Late termination of pregnancies is an issue of grave concern with many practical medical aspects, ethical questions, and professional dilemmas. This is especially of concern because of the viability of the fetus and should only take place in order to prevent harm to the physical and mental health of the mother or due to an anomaly or disability of the fetus.

## BIOSKETCHES

**Isack Kandel**, **MA, PhD,** is Senior Lecturer at the Faculty of Social Sciences, Department of Behavioral Sciences, the Academic College of Judea and Samaria, Ariel. During the period 1985–93, he was Director of the Division for Mental Retardation, Ministry of Labour and Social Affairs, Jerusalem, Israel. E-mail: Kandeli@aquanet.co.il

**Joav Merrick, MD, DMSc,** is Professor of Child Health and Human Development affiliated with the Zusman Child Development Center and Division of Community Health at the Ben Gurion University,

Beer-Sheva, Israel and presently the Medical Director of the Division for Mental Retardation, Ministry of Social Affairs, Jerusalem and the Director of the National Institute of Child Health and Human Development. He has numerous publications in the field of child and human development, rehabilitation, intellectual disability, disability, health, welfare, abuse, advocacy and prevention. Dr. Merrick received the Peter Sabroe Child Award for outstanding work on behalf of Danish Children in 1985 and the International LEGO-Prize ("The Children's Nobel Prize") for an extraordinary contribution towards improvement in child welfare and well being in 1987. E-mail: jmerrick@internet-zahav.net. Website: www.nichd-israel.com
